# Dr. Montgomery Linville

**Published:** 1911-01

**Authors:** 


Dr. Montgomery Linville, of New Castle, Pa., died at his home November 14, 1910, from the effects of poison accidentally self administered, aged 56 years. He was a graduate of Jefferson Medical College, 1873, a member of the American Medical Association, of the National Association of Railway Surgeons, and a Fellow of the American Association of Obstetricians and Gynecologists. He was Attending Surgeon to the Shenango Valley Hospital and Surgeon for several years of the Pennsylvania Railway System, at New Castle.

